# Efficacy and Safety of Urethral Catheter with Continuous Infusion of Ropivacaine after Urologic Surgery: A Pilot Prospective Randomized Controlled Trial

**DOI:** 10.3390/jpm14080835

**Published:** 2024-08-06

**Authors:** Kwang Taek Kim, Myungsun Shim, Kookjin Huh, Sang Hoon Song, Young Jun Uhm, Il Tae Son, Kyung Jin Chung, Dae-Kyung Kwak, Yi Hwa Choi, Hwanik Kim

**Affiliations:** 1 Department of Urology, Gachon University Gil Medical Center, Gachon University School of Medicine, Incheon 21565, Republic of Korea; shinekkt@gmail.com (K.T.K.); kjchung@gilhospital.com (K.J.C.); 2 Department of Urology, Hallym University Sacred Heart Hospital, Hallym University College of Medicine, Anyang 14068, Republic of Korea; grantmsshim@gmail.com (M.S.); janosacs@hallym.or.kr (K.H.); uhm200@naver.com (Y.J.U.); 3 Department of Urology, Asan Medical Center, University of Ulsan College of Medicine, Seoul 05505, Republic of Korea; mdexodus@naver.com; 4 Department of Surgery, Hallym University Sacred Heart Hospital, Hallym University College of Medicine, Anyang 14068, Republic of Korea; 1tae99@hallym.or.kr; 5 Department of Orthopedic Surgery, Hallym University Sacred Heart Hospital, Hallym University College of Medicine, Anyang 14068, Republic of Korea; limitkdk@hallym.or.kr; 6 Department of Anesthesiology and Pain Medicine, Hallym University Sacred Heart Hospital, Hallym University College of Medicine, Anyang 14068, Republic of Korea; pcyhchoi@hallym.or.kr

**Keywords:** analgesic-eluting urethral catheter, bladder discomfort, urethral pain, ropivacaine, prospective randomized controlled trial

## Abstract

Background: Catheter-related bladder discomfort (CRBD) has been found in many patients with urologic surgery. The authors investigated the effect of analgesic-eluting urethral catheters on postoperative CRBD. Methods: 60 subjects scheduled for urologic surgery requiring urethral catheterization were randomized prospectively to one of three groups (control arm, 0.5% ropivacaine 1 mL/h arm [Study 1 arm] and 0.5% ropivacaine 2 mL/h arm [Study 2 arm]; *n* = 20 each). The incidence and severity of CRBD were evaluated postoperatively at 24 h as primary outcomes. The incidence of adverse events regarding urethral catheter utilization was assessed as a secondary outcome. Results: The CRBD incidence at 24 h postoperatively in the control, study 1 and study 2 arms was 50.0%, 10.0%, and 15.0%, respectively (*p =* 0.002). The CRBD severity at 24 h postoperatively showed that patients in the study 1 and study 2 arms had significantly less postoperative CRBD than those in the control arm (visual analog score [VAS]; the mean VAS of the control, study 1, and study 2 arms: 2.1 vs. 1.6 vs. 0.9, *p =* 0.045). Urethral pain regarding catheter was significantly less severe in the study arms than in the control (VAS score: 6.2 vs. 1.5 vs. 1.4, *p* < 0.001). The severity and incidence of adverse events did not differ significantly among groups (*p* = 0.287). Peri-catheter leakage was more frequent in the study 2 arm (*p =* 0.057). Conclusion: The proper usage of a ropivacaine-eluting catheter can not only alleviate CRBD but reduce catheter-related urethral pain in patients with urologic surgery followed by catheterization, without major adverse events.

## 1. Introduction

Indwelling urinary catheters (IUCs) are commonly located in the bladder in patients to help effective urine drainage and prevent urinary retention and renal deterioration [1]. However, up to nearly 90% of catheter users report catheter-related bladder discomfort (CRBD) [2,3]. CRBD is linked to the upregulated inflammation of the bladder and urethra, inducing irritative symptoms related to involuntary bladder smooth muscle contractions caused by the over-innervation of muscarinic receptors in the bladder detrusor muscle [4]. Agents like antimuscarinics, analgesics, or even antibiotics agents are often utilized to relieve CRBD, which might cause unexpected adverse events. One recent review analyzed numerous approaches for CRBD prevention and concluded that dexmedetomidine and gabapentin were ranked best, considering the efficacy and adverse effects of all drugs [5]. A prospective randomized controlled trial (RCT) by Shim et al. reported that the intraoperative administration of ketorolac and magnesium surgery significantly reduced the incidence of early CRBD after the transurethral resection of a bladder tumor [6].

Considering these ambivalent impacts, the topical administration of analgesics might be an effective alternative to alleviate CRBD. Transurethral irrigation with lidocaine towards the end of endoscopic surgery for bladder cancer increased the satisfaction of patient and alleviated the incidence of moderate-to-severe CRBD by over 50%, as shown in an RCT conducted by Singh et al. [7]. Some drug delivery devices have already been designed that can be inserted non-surgically through the urethra [8,9,10]. However, they require a separate procedure for drug administration and are mainly targeted at the bladder, not the urethra. A previous RCT by Imai et al. [11] reported the efficacy of a urethral catheter for men with a local anesthetic 2% lidocaine injection port (Three slits), but did not elucidate significant differences between groups regarding CRBD incidence and only conducted an evaluation from the immediate postoperative period to the post 6 h period.

To control CRBD more effectively and safely, researchers have developed a sustained release of anesthetic IUC with 8–10 pores for analgesics as a solitary device, with drug delivery capabilities targeting both the bladder and urethra. Therefore, we conducted an investigation to verify the efficacy and safety of a self-developed analgesic injection catheter through a multicenter prospective RCT.

## 2. Patients and Methods

### 2.1. Patients and Design

A multi-institutional, prospective, single-blinded randomized controlled pilot study was conducted between April 2023 and December 2023 at 3 medical centers. The inclusion criteria were as follows: (1) male subjects between the ages of 19 and 79, (2) subjects with an Eastern Cooperative Oncology Group performance status of 0 or 1, and (3) a willingness and ability to participate in the trial. Patients were excluded if (1) there were any prior treatments for surgical disease such as radiation therapy, chemotherapy, or hormonal therapy prior to surgery or planned after surgery, (2) there was any history of a previous surgery or radiation therapy in the pelvic cavity, (3) there was any allergic reaction to pain medication, (4) subjects had severe hypertension, or (5) there were any plans for pregnancy, (6) there was any unvoluntary willingness to participate in this study. In addition, patients with inappropriate medical records for analyzing as study subjects by the investigators’ discretion after a thorough review were excluded from the trial. The clinical trial is registered with the Clinical Research Information Service (KCT0008598).

All of the male participants were instructed to respond the international prostate symptom score (IPSS) [12], overactive bladder symptom score (OABSS) [13] a questionnaire, and a two-question survey, evaluating catheter’s preference and discomfort, modified and adapted from a ureteral stent symptom questionnaire [14]. Entire protocols of the study were in accordance with the principles of the Helsinki Declaration after obtaining approval by the Hallym University Sacred Heart Hospital institutional review board (IRB) (IRB number: HALLYM 2023-02-001-001).

### 2.2. Detailed Processes of Study

Subjects were instructed to complete the aforementioned IPSS, the OABSS questionnaire, and the urethral pain score questionnaire prior to urologic surgery while giving informed consent for this study. They were randomized to one of the three arms (control arm, 0.5% ropivacaine 1 mL/h arm [Study 1 arm], and 0.5% ropivacaine 2 mL/h arm [Study 2 arm]). 

**Figure 1:**
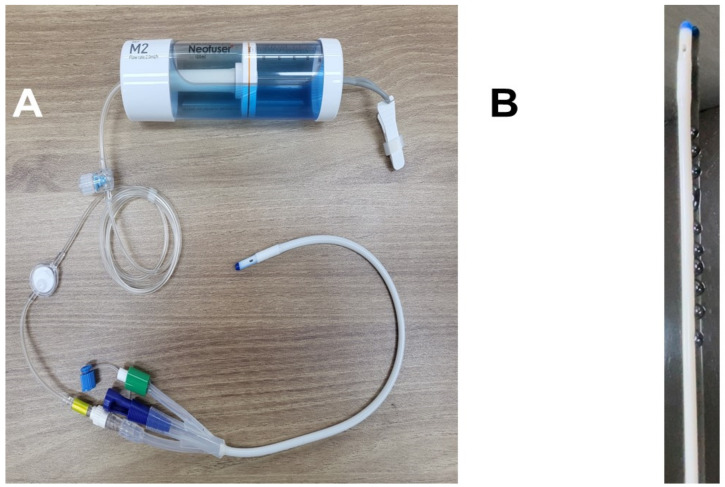
Urethral catheter before use (**A**) and during use (**B**).

Following urologic surgery under general anesthesia that required catheterization, the investigators inserted a 16Fr urethral catheter (trade name: Free-Foley) into the subjects’ urethra (Figure 1A). To prevent spontaneous removal from the bladder, one of the investigators inflated the catheterization tube balloon. Then, the investigators pulled the catheter tube to ensure that the balloon was in close contact with the bladder neck. The agents were then infused at specific concentrations and rates through the drug inlet of the urethral catheter. Then, the agent is delivered noninvasively through a urethral catheter into the lumen of the urethra. If the catheter is considered as a sprinkler hose, the drug is delivered as if it were being sprayed into the luminal wall of the urethra from multiple micro-exits in the hose. The agents used in this study were normal saline at 1 cc/hour, ropivacaine 0.5% at 1 cc/hour (equivalent to 5 mg ropivacaine/hour), or ropivacaine 0.5% at 2 cc/hour (equivalent to 10 mg ropivacaine/hour) (Figure 1B). The catheter remained in the subjects’ bodies for at least 12 h after surgery. Except for when the investigators determined that the postoperative pain was related to the procedure and not to the catheter, no postoperative anesthesia was used. 

At 24 h postoperatively, patients were instructed to complete the same preoperative questionnaires as well as additional questionnaires regarding catheter preference and discomfort. 

### 2.3. Study Endpoints

The primary outcomes were the incidence and severity of CRBD assessed at 24 h postoperatively. A secondary outcome was the incidence of adverse events during Foley catheter utilization among groups.

### 2.4. Sample Size Calculation

As a study to confirm feasibility, secure basic results, safety and efficacy, this study set the reduction effect of the visual analog scale (VAS) score in the study arm compared with the control as the outcome evaluation variable, assuming the difference in VAS score among groups to be 0.56 points. The investigators calculated the required number of subjects using G*Power 3.1.9.7 [15], with a power of 0.95 and significance level of 0.05. A total sample size of 108 subjects (36 subjects per group) was sufficient to reject the null hypothesis of the trial. As this is a pilot study, the investigators evaluated the analgesic-eluting catheter’s effectiveness in half of the study subjects in each group. Finally, we aimed to enroll a total of 60 subjects, considering an expected drop-out rate of 10% (20 subjects per group). 

### 2.5. Randomization of Groups

A random sequence was generated utilizing computer software (http://www.random.org/ (accessed on 1 April 2023)). Participants were allocated into one of the three arms with an allocation ratio of 1:1:1. All medications were packed in same cases and placed in similar opaque containers so that the participants could not be made aware of the group to which they were allocated.

### 2.6. Statistical Analysis

Clinical characteristics were compared between groups according to the drugs and their administration rate using the chi-square test and Fisher’s exact test for categorical variables and a one-way ANOVA test for continuous variables. All tests were two-sided with a value of 0.05 and statistical significance was set at *p* ≤ 0.05 using the IBM Statistical Package for the Social Science Statistics for Windows (SPSS) version 27.0 (IBM SPSS Statistics, IBM Corp., Armonk, NY, USA).

## 3. Results

**Figure 2:**
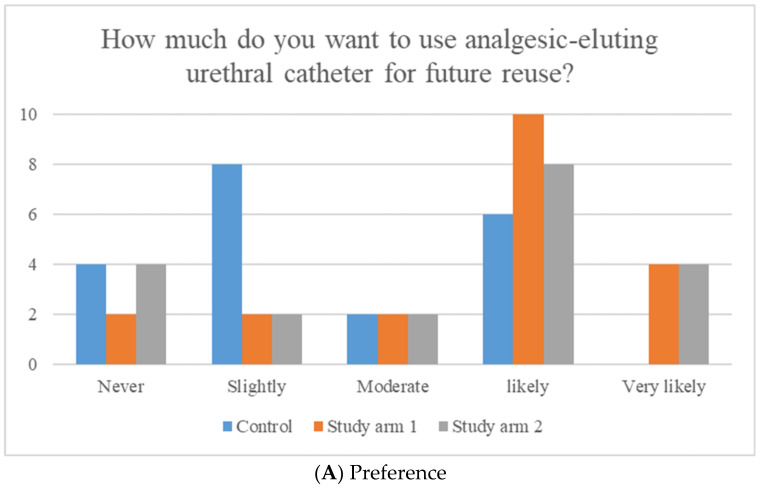

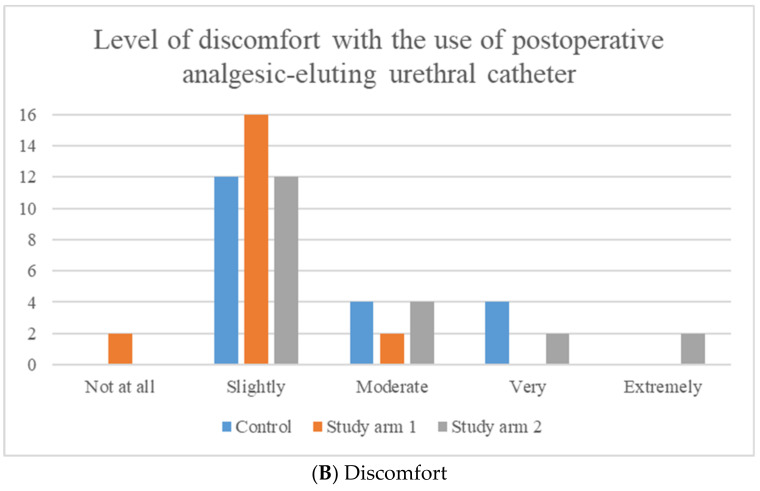
Preference and discomfort with analgesic-eluting urethral catheter use by group. (**A**) Preference (*p* = 0.658). (**B**) Discomfort (*p* = 0.558).

All 60 patients satisfied the study criteria and were included in the final analysis. The subjects’ mean age was 57.4 years old, without significant differences observed among the groups. Insignificant differences were found among the groups regarding the ASA score, the body mass index, the underlying diseases, the type of urologic surgery conducted, the duration of postoperative catheterization, or length of hospital stay (Table 1). When analyzing the pre- and postoperative IPSS and OABSS questionnaire scores, no significant differences were observed among the groups (Table 2). The intention to reuse the catheter in the future was higher in the analgesic-eluting group (both study 1 and 2 groups), which did not reveal statistical significance (*p* = 0.658). Additionally, most subjects, regardless of group, reported slight discomfort with the catheter, without significant differences among the groups (*p* = 0.558). Only two patients in study arm 1 reported no discomfort with the catheter (Figure 2).

The CRBD incidence at 24 h postoperatively in the control, study 1 and study 2 arms was 50.0%, 10.0%, and 15.0%, respectively (*p =* 0.002). The CRBD severity at 24 h postoperatively showed that subjects in both study groups had significantly less postoperative CRBD than those in the control arm (visual analog score [VAS]; the mean VAS of the control, study 1, and study 2 arms: 2.1 vs. 1.6 vs. 0.9, *p =* 0.045). Catheter-related urethral pain was significantly less severe in both study groups than in the control (VAS: 6.2 vs. 1.5 vs. 1.4, *p* < 0.001). Preoperative bladder discomfort and urethral pain did not differ significantly among the groups (Table 2).

The severity and incidence of the adverse events did not differ significantly among the groups (*p* = 0.287). All adverse events corresponded to Clavien–Dindo grade 1. There was more frequent peri-catheter leakage in the study 2 arm compared to the others (*p =* 0.057). There were two subjects with postoperative acute urinary retention (PAUR) (Table 2). The first patient with PAUR underwent both retrograde intrarenal surgery and cystolitholapaxy for multiple renal and bladder stones. From a thorough review of his medical records, the investigators found that he had benign prostatic hyperplasia (prostate size: 86 cc), diabetes mellitus, and a history of multiple surgeries under general anesthesia. All of these factors, including the most recent surgery, could contribute to his PAUR rather than just a drug side effect precipitating PAUR. The second patient with PAUR underwent scrotal hydrocelectomy for hydrocele. As this patient had no underlying disease, his PAUR could be attributed to a drug side effect.

## 4. Discussion

Many efforts made over the years to treat and control CRBD can be easily seen in several RCT studies. Cao et al. [16] reported from their RCT that the intravenous administration of 0.5 µg/kg dexmedetomidine alleviated early postoperative CRBD incidence with minimal adverse events. In detail, the incidence of moderate-to-severe CRBD was significantly lower in the arm using dexmedetomidine 0.5 µg/kg and the arm using dexmedetomidine 1 µg/kg than in the placebo arm at 0 h, 1 h, and 6 h postoperatively. Compared with the baseline, both the heart rate and mean arterial pressure were significantly decreased in the group administered dexmedetomidine 1 µg/kg at 1 min and 30 min after extubation. Jee et al. [17] concluded from their RCT of 76 participants who underwent ureteroscopic stone removal that chlorpheniramine maleate injection before the induction of anesthesia had a minimal effect on the severity and incidence of postoperative CRBD, but it lessened the usage of tramadol required to control postoperative CRBD. They reported that the incidence of moderate CRBD was increased in the control than in the group using only chlorpheniramine maleate at 0 h (26.3% vs. 5.3%, *p* = 0.025). Moreover, fewer patients in the group with chlorpheniramine maleate needed rescue tramadol to alleviate postoperative CRBD (31.6% vs. 60.5%, *p* = 0.011). Kim et al. [18] proved the effect of intravenous lidocaine for CRBD prevention after transurethral surgery from an RCT of 132 patients. They reported that the incidence of moderate-to-severe CRBD was significantly decreased in the group with intravenous lidocaine than in the placebo group (1 h: 10.6% vs. 27.3%, *p* = 0.026; 2 h: 0.0% vs. 15.2%, *p* = 0.003). Significantly higher patient satisfaction was shown in the arm with intravenous lidocaine than in the control arm (5.0 vs. 4.0, *p* < 0.001). Our results of CRBD incidence and severity are comparable to these studies [16,17,18].

Predictors of CRBD have been reviewed in the literature. Binhas et al. [19] reported that male gender (OR = 3.2, *p* < 0.06) was an independent predictor of moderate and severe CRBD. Li et al. [20] concluded that open abdominal surgery (OR = 3.074, *p* < 0.05) and the experience of catheterization (OR = 2.458, *p* < 0.05) were related with moderate and severe CRBD. A literature review conducted by Mitobe et al. [21] analyzed various factors (patient, surgical, device and insertion technique and anesthetic factors). They reported that the history of urethral catheterization, experiences of urological, obstetrical and gynecological surgery, a urinary catheter size 18 Fr or larger, the lack of postoperative analgesics, and the lack of catheter lubrication were predictors of CRBD. A recent prospective study conducted by Liang et al. [22] developed a nomogram for predicting CRBD presence but not severity. The nomogram included six variables as follows: the type and duration of surgery, male gender, oxycodone, dexmedetomidine, and the time of catheterization. Nevertheless, the size of the urinary catheter was not included due to the center’s specific restriction, which might be a limitation of the study. Its areas under the receiver operating characteristic curve were 0.78 in the original dataset and 0.759 after bootstrapping. 

Ropivacaine was selected as the catheterized analgesic for this study due to its reduced cardiotoxicity compared to lidocaine or bupivacaine [23]. Moreover, its efficacy is known to be nearly equivalent to lidocaine [24], and its ability to control postoperative pain for a relatively long period without the need for additional infusion compared to lidocaine has also been reported [19]. One Dutch prospective study [25], which could support our findings, elucidated that periurethral and perivesical administrations with ropivacaine have the ability to decrease the incidence of early postoperative CRBD by up to 49% in patients with robot-assisted laparoscopic radical prostatectomy. They also reported that patients treated with perivesical and/or periurethral administrations revealed significantly fewer incidences of CRBD compared to the control patients (46.5% vs. 60.7%, *p* = 0.001). One of our study’s strengths is that we found that even in patients taking oral analgesics, the use of Free-Foley catheters was associated with significantly less pain, significantly less CRBD, and significantly higher satisfaction with use compared with conventional catheters. Another strength is that it is the first RCT to demonstrate that the application of a proprietary, indigenously patented technology with a catheter to deliver a constant hourly dose of analgesics in multiple directions into the urethra enables reduced CRBD along with reduced urethral pain. Moreover, the study elucidated that there was no significant difference in the incidence of voiding-related complications, including acute urinary retention, among the groups.

Nevertheless, our study has several limitations. One limitation of the trial is the relatively small number of subjects despite its multicenter prospective design. This may be attributed to the unfamiliarity of this trial process and lack of patient comprehension as similar studies are not commonly conducted domestically. We could not avoid investigators’ selection bias due to the feature of a single-blind study, although the main investigators did not actually recognize who was in each specific group. Furthermore, the study did not examine the predictors of CRBD and urethral pain. Patients might not be able to distinguish between CRBD pain and surgical pain. Our pain assessment investigators could not provide an explanation for this, and therefore, we could have investigated both parameters. A further limitation is that the investigators enrolled only male patients who had undergone urological surgery, including ureteroscopy and other endourological procedures that may require the use of various types of ureteral stents. The presence of these stents may contribute to the development of urethral pain and related symptoms, which could potentially impact the findings of this study. Future study is warranted to see if these results are reproduced in a cohort with different surgeries. Moreover, we could not evaluate the effect of urethral catheter size because we consistently applied one size of the catheter to every subject. Finally, pain managements with analgesics were inconsistent due to the surgeon’s discretion and each center’s individuality. 

## 5. Conclusions

A urethral catheter with the continuous infusion of a proper dose of ropivacaine could not only reduce the incidence or severity of CRBD but also alleviate catheter-related urethral pain in patients undergoing urologic surgery with insignificant adverse events. The results of this study may provide evidence for its use in men at risk of developing CRBD. Further studies could focus on relieving postoperative CRBD and pain, evaluating other cohorts like female cohorts undergoing general or gynecological surgeries.

## Figures and Tables

**Table 1:** Baseline characteristics.

	Control Arm	Study Arm 1	Study Arm 2	*p* Value
	(*n* = 20)	(*n* = 20)	(*n* = 20)	
Age (year)	60.6 ± 9.5	54.8 ± 10.0	56.9 ± 13.5	0.509
ASA score 1–2	16 (80.0%)	16 (80.0%)	16 (90.0%)	1.000
3	4 (20.0%)	4 (20.0%)	4 (20.0%)	
Body mass index	23.8 ± 4.8	25.5 ± 3.9	25.4 ± 4.3	0.616
Diabetes mellitus	2 (10.0%)	4 (20.0%)	4 (20.0%)	0.787
Hypertension	12 (60.0%)	8 (40.0%)	10 (50.0%)	0.670
Previous catheter history	10 (50.0%)	6 (30.0%)	8 (40.0%)	0.659
Operation procedure				0.627
Ureteroscopic stone surgery	10 (50.0%)	12 (60.0%)	16 (80.0%)	
Scrotal surgery	4 (20.0%)	4 (20.0%)	0 (0.0%)	
Renal surgery	6 (30.0%)	4 (20.0%)	4 (20.0%)	
Length of stay (day)	2.6 ± 2.4	3.0 ± 2.9	1.6 ± 1.3	0.387
Length of postoperative catheterization (day)	1.3 ± 0.7	1.3 ± 0.5	1.0 ± 0.0	0.287

Values are presented as mean ± standard deviation.

**Table 2:** Efficacy and complication profile.

	Control Arm	Study Arm 1	Study Arm 2	*p* Value
	(*n* = 20)	(*n* = 20)	(*n* = 20)	
CRBD	10 (50.0%)	2 (10.0%)	3 (15.0%)	0.002
Bladder discomfort score (pre-catheterization) (VAS)	0.4 ± 0.5	0.5 ± 0.7	0.5 ± 0.5	0.909
Bladder discomfort score (post catheterization) (VAS)	2.1 ± 1.0	1.6 ± 1.2	0.9 ± 0.9	0.045
Complication rate (all Clavien–Dindo Gr 1)	10 (50.0%)	4 (20.0%)	10 (50.0%)	0.287
Peri-catheter leakage	2 (10.0%)	2 (10.0%)	10 (50.0%)	0.057
Acute urinary retention	0 (0%)	2 (10.0%)	0 (0%)	0.355
Urethral pain VAS score (pre-catheterization, Pre)	0.4 ± 0.5	0.4 ± 0.5	0.5 ± 0.5	0.885
Urethral pain VAS score (post catheterization, Post)	6.2 ± 2.2	1.5 ± 0.8	1.4 ± 1.0	<0.001
Total IPSS score (Pre)	9.0 ± 4.3	7.4 ± 6.2	9.2 ± 10.3	0.838
Total IPSS score (Post)	9.1 ± 4.4	7.6 ± 5.9	10.9 ± 9.9	0.592
IPSS QoL score (Pre)	3.2 ± 1.0	2.6 ± 1.2	2.7 ± 1.8	0.591
IPSS QoL score (Post)	3.2 ± 1.0	2.7 ± 1.4	3.1 ± 1.6	0.692
Total OABSS score (Pre)	4.2 ± 1.8	3.2 ± 2.8	3.2 ± 3.2	0.629
Total OABSS score (Post)	4.4 ± 2.0	3.4 ± 3.0	3.5 ± 3.3	0.687

Values are presented as mean ± standard deviation.

## Data Availability

The datasets used and/or analyzed during the current study are available from the corresponding author upon reasonable request.
